# *Qing*-*dai* powder promotes recovery of colitis by inhibiting inflammatory responses of colonic macrophages in dextran sulfate sodium-treated mice

**DOI:** 10.1186/s13020-015-0061-x

**Published:** 2015-10-13

**Authors:** Hai-Tao Xiao, Jiao Peng, Dong-Dong Hu, Cheng-Yuan Lin, Bin Du, Siu-Wai Tsang, Ze-si Lin, Xiao-Jun Zhang, Feng-Ping Lueng, Quan-Bin Han, Zhao-Xiang Bian

**Affiliations:** School of Chinese Medicine, Hong Kong Baptist University, Hong Kong, Hong Kong; School of Pharmacy, Guiyang Medical University, Guiyang, 550004 China; Department of Surgery, LKS Faculty of Medicine, The University of Hong Kong, Hong Kong, Hong Kong; School of Chinese Medicine, Shanghai University of Chinese Medicine, Shanghai, 200030 China; School of Fundamental Medical Science, Guangzhou University of Chinese Medicine, Guangzhou, 510006 China; School of Chinese Medicine, Guangzhou University of Chinese Medicine, Guangzhou, 510006 China

## Abstract

**Background:**

*Qing*-*dai* powder (QDP), comprising *Indigo naturalis* (*Qing*-*dai*) and dried alum (*Ku*-*fan*), was used in Chinese medicine to treat the conditions associated with mucosal hemorrhage, such as ulcerative colitis (UC). This study aims to investigate the effects and potential mechanism of QDP on dextran sulfate sodium (DSS)-induced acute colitis in mice and to examine the regulatory effects of QDP on macrophages.

**Methods:**

Seven- to eight-week-old male C57BL/6 mice were challenged with 2.0 % DSS in drinking water for 5 days and then the colitic mice were arbitrarily allocated into five groups (n = 10 for each group). QDP (0.77, 1.54 and 3.08 g/kg) and sulfasalazine (SASP) (0.20 g/kg) were orally administered for 7 days. The disease activity index was determined by scores of body weight loss, diarrhea and rectal bleeding; histological signs of damage was analyzed by H&E staining; myeloperoxidase activity was measured by colorimetric method, levels of proinflammatory cytokines were determined by ELISA; changes in macrophages in the colon were analyzed by immunohistochemistry (IHC) and flow cytometry. Lipopolysaccharide (LPS)-induced RAW264.7 cells were treated with or without QDP, then the production of TNF-α and IL-6 were measured by ELISA; and protein molecules such as COX-2, iNOS, IкB-α were determined by Western blot.

**Results:**

Oral administration of QDP at dosages of 1.54 and 3.08 g/kg significantly reduced disease activity index on day 12 (*P* < 0.001 for 1.54 g/kg and *P* < 0.0008 for 3.08 g/kg), colon shortening (*P* = 0.012 for 1.54 g/kg, *P* = 0.001 for 3.08 g/kg), histological damage (*P* < 0.001 for 1.54 g/kg, *P* < 0.001 for 3.08 g/kg) and colonic myeloperoxidase activity (*P* = 0.002 for 1.54 g/kg, *P* < 0.001 for 3.08 g/kg) of DSS-treated mice. Moreover, QDP treatment (1.54 and 3.08 g/kg) significantly decreased DSS-induced infiltration of macrophages, and production of TNF-α (*P* = 0.005 for 1.54 g/kg, *P* = 0.002 for 3.08 g/kg), IL-1β (*P* = 0.008 for 1.54 g/kg, *P* = 0.002 for 3.08 g/kg) and IL-6 (*P* = 0.011 for 1.54 g/kg, *P* = 0.004 for 3.08 g/kg) in colonic tissues, and also reduced serum MCP-1 levels (*P* = 0.001 for 1.54 g/kg, *P* < 0.001 for 3.08 g/kg). In RAW264.7 cells, QDP significantly suppressed LPS-induced production of TNF-α and IL-6 (Both *P* < 0.001 for 1.0 μg/mL QDP treatment) and expression levels of COX-2 (*P* = 0.002 and *P* = 0.001 for 1 and 3 μg/mL QDP treatment, respectively) and iNOS (*P* < 0.001 for 3 μg/mL QDP treatment) by inhibiting IкB-α degradation (*P* = 0.007 and *P* = 0.004 for 1 and 3 μg/mL QDP treatment, respectively) and NF-кB p65 nuclear translocation.

**Conclusion:**

QDP suppressed the inflammatory responses of colonic macrophages in DSS-induced UC in mice and LPS-induced RAW264.7 cells.

## Background

Ulcerative colitis (UC), a subtype of inflammatory bowel disease (IBD), is a chronic uncontrolled inflammatory condition of the intestinal mucosa [1], progressively increasing in the incidence rates worldwide [2, 3]. The refractory relapses are due to lack of effective treatment [4]. Current medical therapies for UC mainly focus on inducing remission and preventing relapse [5]. Aminosalicylates [sulfasalazine (SASP) and 5-aminosalicylic acid (5-ASA)], corticosteroid (prednisolone) and thiopurines (azathioprine and 6-mercaptopurine) are commonly recommended as the standard treatments for UC patients [6]. In addition, infliximab and tacrolimus have been used for treating UC patients since 1998 and 2006, respectively [7].

IBD is subject to environmental factors, genetic determinants, microbial exposures and immunoregulatory defects [8]. IBD develops when an excessive immunological reaction in the mucosal immune system respond towards luminal antigens such as dietary factors, commensal bacteria or both in genetically susceptible hosts [9]. Innate immunity is involved during the onset and in the regulation of the severity of IBD. In the development of IBD, the innate immune response increased levels of proinflammatory cytokines and chemokines, including TNF-α, IL-1β and IL-6, exaggerating adaptive immune responses, and resulting in tissue injury and clinical symptoms [10, 11].

A significant increase in the number of macrophages was observed in the inflamed tissue and peripheral blood vessels of IBD patients [12, 13]. During the development of acute colitis in animal models, accumulated macrophages were also observed in the inflamed gut [14, 15]. Interleukins or growth factors at sites of inflammation activated clusters of macrophages, which subsequently secrete a variety of proinflammatory cytokines, resulting in persistent intestinal inflammation [16]. Depletion of macrophages in dextran sulfate sodium (DSS)-induced colitis in mice halted the development of colitis [17]. Thus, suppressing intestinal macrophages could be a promising therapeutic approach to treating IBD.

*Qing*-*dai* powder (QDP) is a herbal medicinal formula comprising *Indigo naturalis* (*Qing*-*dai*) and dried alum (*Ku*-*fan*), for treating hematemesis and hemorrhinia [18]. QDP and its modified formulas are clinically used to treat colitis in China [19–21]. From 2009 to 2013, we at Hong Kong Baptist University Mr. and Mrs. Chan Hon Yin Chinese Medicine Specialty Clinic and Good Clinical Practice Centre prescribed QDP to patients with intractable UC in clinical practice who had failed to respond to treatment with 5-ASA, prednisolone or infliximab. We gave QDP at a dosage of 7.5 g/day (loaded in capsules, orally, twice a day) for 1–2 weeks. There was a study on indirubin 3′-monoxime, a derivative compound of the active component indirubin isolated from *I. naturalis*, observed the compound significantly inhibited the production of pro-inflammatory mediators induced by LPS in RAW264.7 cells through down-regulating NF-кB and JNK signaling pathways [22]. The regulation of macrophage function could be involved in the effect of QDP. The effects and potential mechanism of QDP has not yet been assessed in animal studies.

This study aims to investigate the effects and potential mechanism of QDP on DSS-induced acute colitis in mice and to examine the regulatory effects of QDP on macrophages.

## Methods

### Materials

DSS (molecular weight: 36,000–50,000 Daltons) was purchased from MP Biologicals (Santa Ana, USA). Dimethylsulphoxide (DMSO), lipopolysaccharide (LPS) (Escherichia coli Serotype 055:B5), sulfasalazine (SASP) (purity ≥ 98 %), hexadecyltrimethylammonium bromide, hematoxylin, eosin, *O*-dianisidine dihydrochloride, protease inhibitor cocktails and hydrogen peroxide were purchased from Sigma-Aldrich (St, Louis, MO, USA). Fetal bovine serum, l-glutamine, penicillin, streptomycin and RPMI 1640 cell culture medium were purchased from Invitrogen (Carlsbad, CA, USA). Antibodies against COX-2, iNOS, IκB α and p65 and β-actin were supplied from Cell Signaling Technology, Inc. (Beverly, MA, USA). TNF-α, IL-1β, and IL-6 ELISA kits were purchased from eBioscience (San Diego, CA, USA). Cy5.5 PerCP anti-mouse CD11b, FITC anti-mouse F4/80 were purchased from BD Pharmingen (San Diego, CA, USA). FITC-conjugated donkey anti-rabbit IgG antibody was purchased from Santa Cruz Biotechnology (Santa Cruz, CA, USA). WesternBright™ ECL was supplied from Advansta (Menio Park, CA, USA).

### Preparation of QDP

The ratio of *I. naturalis* and dried alum in QDP is 2:1. Two Chinese medicinal raw herbs were obtained from the dispensary of Traditional Chinese Medicine, Clinical Division, School of Chinese Medicine, Hong Kong Baptist University, Hong Kong. These two Chinese medicinal materials were authenticated by Dr. Hu-Biao Chen (School of Chinese Medicine, Hong Kong Baptist University, Hong Kong) according to the Chinese Pharmacopoeia (version 2010) and other references [23–25]. Voucher specimens (nos. TCM-0110-Q01, TCM-0110-Q02) are stored in our Research Laboratory, School of Chinese Medicine, Hong Kong Baptist University, Hong Kong. Two herbs were mixed, powdered to homogeneous size in a mill and sieved through a 120-mesh filter. Each sample was exactly weighed, ultrasonically extracted with DMSO in a KQ-2200DB ultrasonic cleaner bath (Kunshan Ultrasound Instrument Co., Ltd., Jiangsu, China), and filtered through a syringe filter for subsequent UPLC-QTOF-MS analysis and cell culture treatment. For animal study, QDP was freshly suspended in 0.5 % sodium carboxymethylcellulose (CMC-Na) in distilled water prior to oral feeding to mice.

### Ultra-performance liquid chromatography quadrupole time-of-flight mass spectrometry (UPLC-QTOF-MS) analysis

The components in QDP were identified by UPLC-QTOF-MS. Chromatographic separation was performed by an Agilent 1290 Infinity UPLC system (Santa Clara, CA, USA), equipped with a binary solvent delivery system, a standard auto-sampler and photodiode array detectors (DAD). A 100 mm × 2.1 mm ACQUITY BEH C_18_ 1.7-µm column (Waters Corp., Milford, MA, USA) was used to separate the components of QDP. The mobile phase consisted of (A) 0.1 % formic acid in water and (B) 0.1 % formic acid in acetonitrile. A linear gradient was optimized as follows (flow rate, 0.40 mL/min): 0–2.5 min, 2–5 % B; 2.5–10 min, 5–35 % B; 10–20 min, 35–75 % B; 20–23 min, 75–100 % B; 23–26 min, 100 % B; 26–26.1 min, 100–2 % B; 26.1–30 min, 2 % B. The injection volume was 2 μL and the column temperature was maintained at 40 °C in each run. Mass spectrometry was performed by an Agilent 6540 ultra-high definition (UHD) QTOF mass spectrometer, equipped with a Jet Stream electrospray ionization (ESI) source. Parameters for the Jet Stream technology were set with the superheated nitrogen sheath gas temperature at 350 °C and with a flow rate at 10 L/min. ESI conditions were set as follows: negative ion mode, capillary 4500 V, nebulizer 1.85685 × 10^6^ kPa, drying gas 8 L/min, gas temperature 300 °C, nozzle voltage 300 V, skimmer voltage 65 V; octapole RF peak 600 V, fragmentor 175 V. Mass spectra were recorded across the range *m*/*z* 100–1700 with accurate mass measurement of all mass peaks. A sprayer with a reference solution was used for continuous calibration in negative ion mode with reference masses at *m*/*z* 112.9856 and 966.0007. The full-scan and MS/MS data were processed with Agilent Mass Hunter Workstation software (version B.02.00) (Santa Clara, CA, USA).

### Cell culture

RAW264.7 murine macrophage cells were obtained from the American Type Culture Collection (ATCC No. TIB-71). The cell line was cultured in RPMI 1640 cell culture medium supplemented with 10 % (v/v) fetal bovine serum, 2 mM l-glutamine, 100 U/mL penicillin G and 100 μg/mL streptomycin. The cells were incubated in a humidified 5 % CO_2_ incubator at 37 °C.

### Animals

Seven to eight-week-old male C57BL/6 mice weighing 20–24 g were purchased from the Laboratory Animal Services Center, The Chinese University of Hong Kong. The animals were fed a standard rodent diet with free access to water, and were kept in rooms maintained at 21–23 °C with a 12 h light/dark cycle following international recommendations. All experimental protocols were approved by the Animal Ethics Committees of Hong Kong Baptist University, in accordance with “Institutional Guidelines and Animal Ordinance” (Department of Health, Hong Kong Special Administrative Region).

### Induction of colitis and treatment

Acute colitis was induced by oral administration of 2.0 % (w/v) DSS dissolved in drinking water, for 5 days according to Wirtz et al. [26]. Mice of each experimental group were monitored every day to confirm that they consumed equal volumes of DSS-containing water.

Two sets of experiments were performed. For the first one, 50 colitic mice were arbitrarily allocated into 5 groups: DSS model group, sulfasalazine (SASP, positive reference agent)-treated group, and three QDP-treated groups (n = 10). A vehicle control group with nine normal mice received drinking water without DSS throughout the entire experimental period. Consistent with clinical treatment, QDP was administrated orally to colitic mice at doses of 0.77, 1.54 or 3.08 g/kg/day, comparable with the clinical dosages used in human UC patients. SASP was used as a positive reference agent and it was given at 0.20 g/kg/day according to Kim et al. [27]. The gavage volume was 0.4 mL.

For the second set of experiments to immunophenotype colonic macrophages in colonic lamina propria, twelve colitic mice were arbitrarily allocated into 2 groups: DSS model group (n = 6) and 1.54 g/kg QDP-treated group (n = 6). A vehicle control group of 5 normal mice received drinking water only during the entire experimental period. Both SASP and QDP were dissolved in 0.5 % sodium carboxymethylcellulose (CMC-Na) solution and administrated orally to the mice for 7 days after the onset of colitis. The vehicle control group and DSS model group were fed with 0.4 mL of 0.5 % CMC-Na solution instead of SASP or QDP.

### Evaluation of disease activity index (DAI)

Body weight, stool consistency and rectal bleeding were recorded daily. The DAI was determined by combining the scores of (1) body weight, (2) stool consistency and (3) rectal bleeding [28].

### Histological analysis

Colon tissues were harvested and fixed in 4.0 % paraformaldehyde. Tissue sections were prepared by conventional tissue processing methods, stained with hematoxylin and eosin (H&E), and examined under the light microscope. Colonic damage was assessed as described previously [28].

### Determination of colonic myeloperoxidase (MPO) activity

MPO activity was measured as described in our previous study [28]. One unit of MPO activity was defined as the amount of enzyme present that produced a change in optical density of 1.0 U/min at 25 °C in the final reaction volume. The results were normalized to equal protein levels and quantified as units/mg protein.

### Immunohistochemical analysis

Colonic tissues were fixed in 4.0 % buffered paraformaldehyde, embedded in paraffin and sectioned into 5-μm-thick slices. Sectioned samples were deparaffinized in xylene, rehydrated in a series of graded alcohol, and subjected to antigen retrieval. Antigens were retrieved by incubation with protease K solution (1: 100) for 20 min.

Endogenous peroxidase was quenched with 3.0 % hydrogen peroxide in methanol for 30 min. Sections were further blocked with 3.0 % bovine serum albumin (BSA) in PBS, exposed to 0.5 % Triton X-100 for 1 h for reducing nonspecific antibody binding and incubated with mouse F4/80 antibody (AbD serotec, Raleigh, North Carolina, USA) at 4 °C overnight. The sections were washed with PBS three times, incubated with biotinylated anti-rabbit immunoglobulins, followed by peroxidase-labeled streptavidin, and 3, 3′-diaminobenzidine chromogen substrate added to make antibody binding visible according to the protocol of the LSAB kit (LSAB-DAKO, Copenhagen, Denmark). Sections were then washed with PBS and counterstained with hematoxylin. After dehydration with a series of increasingly concentrated ethanol, sections were mounted with neutral gum. Five random fields at 400× magnification were counted in each sectioned sample by a researcher blinded to the treatment. The number of macrophages per μm^2^ of mucosa was quantified by the Image J software (National Institutes of Health, Bethesda, Maryland, USA).

### Immunophenotyping of colonic macrophages

Colonic lamina propria cells were isolated as previously described with slight modifications [29, 30]. The colons were removed from mice immediately after euthanasia. Subsequently, the colonic tissues were immersed in DMEM containing 100 U/mL of penicillin and 100 μg/mL of streptomycin, dissected longitudinally and cut into 0.5–1-cm pieces. Then, intestinal epithelial cells were dissociated by incubation with Ca^2+^ and Mg^2+^-free HBSS containing 5 mM EDTA and 1 mM DTT for 20 min at 37 °C, twice. After thorough washing with PBS, colonic tissues were then incubated with digestion buffer (RPMI 1640 containing 3 mg/mL dispase II, 0.5 mg/mL collagenase D, and 0.5 mg/mL DNase I) for 30 min at 37 °C, twice. Finally, the cells were labeled with anti-CD11b and anti-F4/80 antibodies, and analyzed by flow cytometry. Appropriate isotype-matched IgGs were used as negative controls.

### Enzyme-linked immunosorbent assay (ELISA) analysis

The cytokines TNF-α, IL-1β and IL-6 in the culture supernatants of RAW264.7 cells and colonic tissues and the chemokine monocyte chemoattractant protein-1 (MCP-1) in serum samples were measured with TNF-α, IL-1β, IL-6 and MCP ELISA kits, respectively (eBioscience, San Diego, CA, USA) according to the manufacturer’s protocols.

### Western blot analysis

After treatment with a range of concentrations of QDP (0.3–3 μg/mL) in the presence or absence of 1 μg/mL LPS, RAW264.7 cells were analyzed by immunoblotting as described previously [31]. Primary antibodies that recognize iNOS, COX-2 and IκB-α were used, and specific proteins were detected by WesternBright™ ECL. Equal loading was assessed by a β-actin antibody, and the amount of total protein present was normalized to the level of β-actin.

### Cellular localization of nuclear factor kappa B (NF-κB) p65

RAW264.7 cells were cultured directly on glass cover slips in a 35-mm dish for 24 h and pretreated with QDP for 1 h in the presence or absence of 1 μg/mL LPS. Cells were fixed with 4 % paraformaldehyde, treated with 0.2 % Triton X-100, and blocked with 3 % BSA. Subsequently, cells were incubated with a rabbit anti-NF-κB p65 polyclonal antibody (1:100) at 4 °C overnight. After extensive washing with PBS, cells were further incubated with a secondary FITC-conjugated donkey anti-rabbit IgG antibody (1:100) for 1 h at room temperature. Nuclei were counterstained with DAPI solution (1 μg/mL), and cells were analyzed by a fluorescence microscope (Carl Zeiss, Oberkochen, Germany).

### Statistical analysis

The data were presented as mean ± standard deviation (SD). Statistical differences among groups were evaluated by one-way analysis of variance (ANOVA) and Duncan’s multiple range test by SPSS 16.0 statistical software (SPSS Inc., Chicago, IL, USA). *P* values <0.05 were considered statistically significant. Dose-dependence was visually determined.

## Results

### Identification of chemical components of QDP

**Fig. 1:**
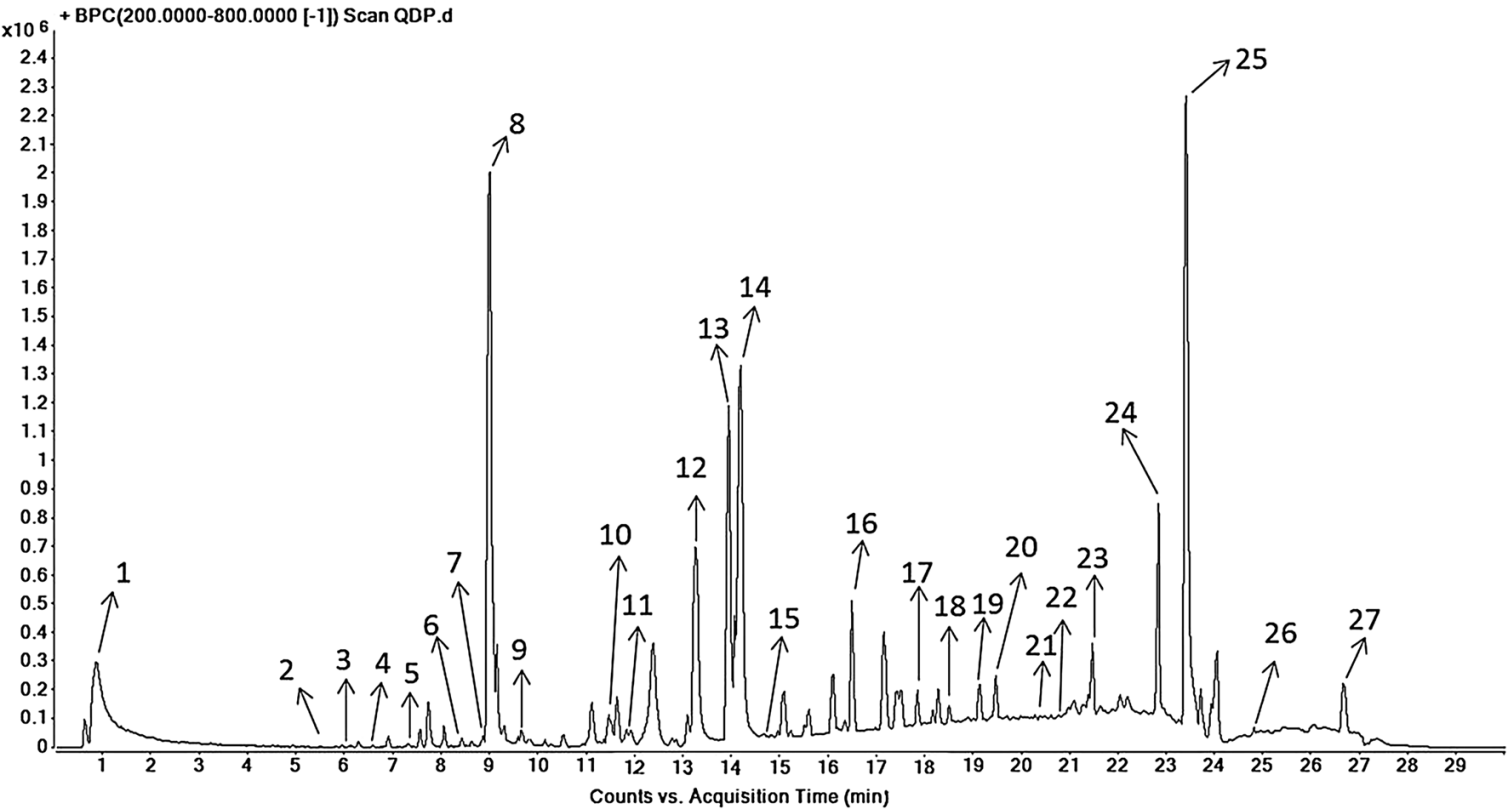
Elementary particle flow graph (BPI) chromatogram monitored in positive ion mode for QDP

Under the optimized conditions [32], the major components in QDP were well separated and detected within 30 min (Table 1). Taking retention time (tR), *m*/*z*, ultraviolet (UV) absorption characteristics (lambda max), and relevant references into consideration, 27 peaks representing individual chemical components were identified. The representative chromatograms, monitored by DAD and MS, are shown in Fig. 1.

**Table 1:** Main components of QDP

Peak	tR (min)	Assigned identity	Molecular formula
1	0.999	Butanedioic acid	C_4_H_6_O_4_
2	5.775	Isatin	C_8_H_5_NO_2_
3	5.908	2,3-Dihydro-4-hydroxy-2-oxo-1H-indole-3-acetic acid	C_10_H_8_N_2_O_2_
4	6.511	Deoxyvascinone	C_11_H_10_N_2_O
5	7.729	10H-indolo [3, 2-b] quinoline	C_15_H_10_N_2_
6	8.436	2,3-Dihydro-3,4-dihydroxy-2-oxo-1H-indole-3-acetonitrile, 3-Cyanomethyl-3,4-dihydroxyoxindole	C_10_H_8_N_2_O_3_
7	8.856	3-(2-Hydroxyphenyl)-4(3H)-quinazolinone	C_14_H_10_N_2_O_2_
8	8.995	10H-indolo[3,2-b]quinoline-11-carboxylic acid amide	C_16_H_11_N_3_O
9	9.589	Tryptanthren	C_15_H_8_N_2_O_2_
10	11.515	Syringin	C_17_H_24_O_9_
11	11.909	3-(2-Carboxyphenyl)-4(3H)-quinazolinone	C_15_H_10_N_2_O_3_
12	13.240	Indigo	C_16_H_10_N_2_O_2_
13	13.972	Indican	C_14_H_17_NO_6_
14	14.185	Indirubin	C_16_H_10_N_2_O_2_
15	14.658	2-[Cyano(3-indolyl)methylene]-3-indolone	C_18_H_11_N_3_O
16	16.612	8,11,12-Trihydroxy-9-octadecenoic acid	C_19_H_36_O_5_
17	17.883	Anthranilic acid	C_7_H_7_NO_2_
18	18.492	Salicylic acid	C_7_H_6_O_3_
19	19.161	Octadecanoic acid	C_18_H_36_O_3_
20	19.343	Bisindigotin	C_32_H_18_N_4_O_2_
21	20.550	Qingdainone	C_23_H_13_N_3_O_2_
22	20.854	Betulin	C_30_H_50_O_2_
23	21.463	Sugiol	C_20_H_28_O_2_
24	22.801	Rotenol	C_23_H_24_O_6_
25	23.417	Sitosterol	C_32_H_53_N_7_O_2_
26	24.686	Daucosterol	C_35_H_60_O_6_
27	26.836	Clerosterol	C_29_H_48_O

### QDP attenuates the severity of DSS-induced colitis

**Fig. 2:**
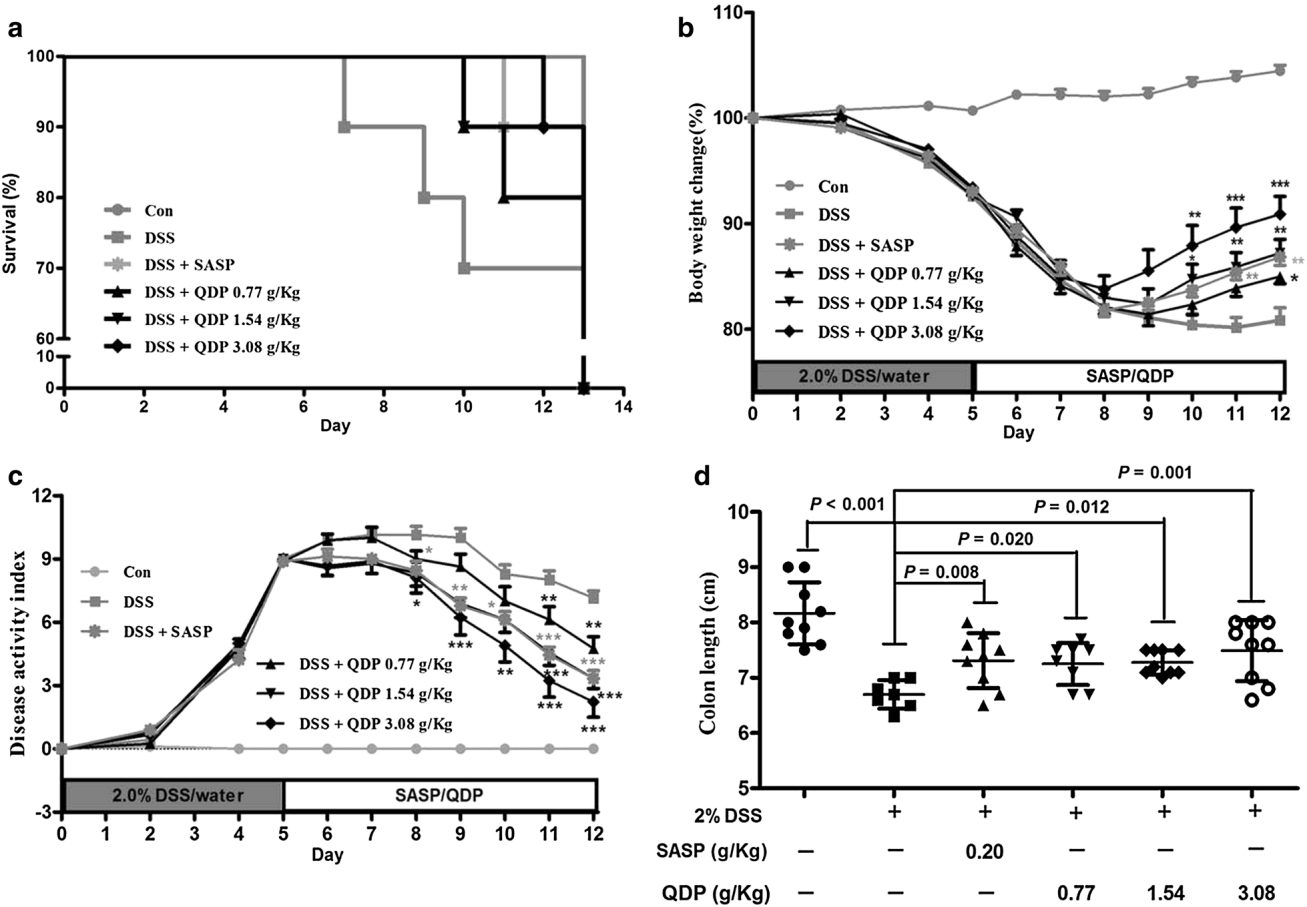
Effects of QDP on mortality **a** body weight change; **b** disease activity index; **c** and colon length; and **d** of mice with DSS-induced colitis. Colitis was induced in all groups except control group. QDP and SASP were administered to mice from day 6 to 12. The change in body weight was taken as the difference between the body weight before induction of colitis and that immediately before sacrifice on day 13. Disease activity index was determined by combining scores of (1) body weight loss; (2) stool consistency; and (3) stool blood. On day 13, mice were sacrificed, and colon length was measured. Survival data of challenged mice were collected from 9 to 10 mice respectively, and other data were expressed as mean ± SD (n = 7–9). ^###^ *P* < 0.001, compared with control group; *P < 0.05, **P < 0.01 and ***P < 0.001, compared with DSS model group

Oral administration of 2.0 % DSS in drinking water resulted in high mortality; up to 30.0 % of the total mice in the DSS model group died. QDP treatment could reduce the mortality rate of DSS-treated mice, to 20.0, 10.0 and 10.0 % for low-, medium- and high-dose QDP-treated groups, respectively (Fig. 2a). Starting from day 5, DSS resulted in rapid loss of body weight and serious clinical disease symptoms (diarrhea and occult fecal blood) in C57BL/6 J mice, and lasted 3–4 days. After that, mice began to recover. QDP treatment improved body weight recovery and DAI of DSS-treated mice, particularly in the medium- and high-dose QDP treatment groups when compared with the DSS model group (both *P* < 0.001) (Fig. 2b, c). In addition, the DSS-induced model of colitis is associated with a marked decrease in colon length [28]. Treatment with QDP significantly prevented colon shortening in a dose-dependent manner (*P* = 0.020, *P* = 0.012, *P* = 0.001, respectively) (Fig. 2d).

### QDP decreases colonic tissue damage and reduces colonic MPO activity

**Fig. 3:**
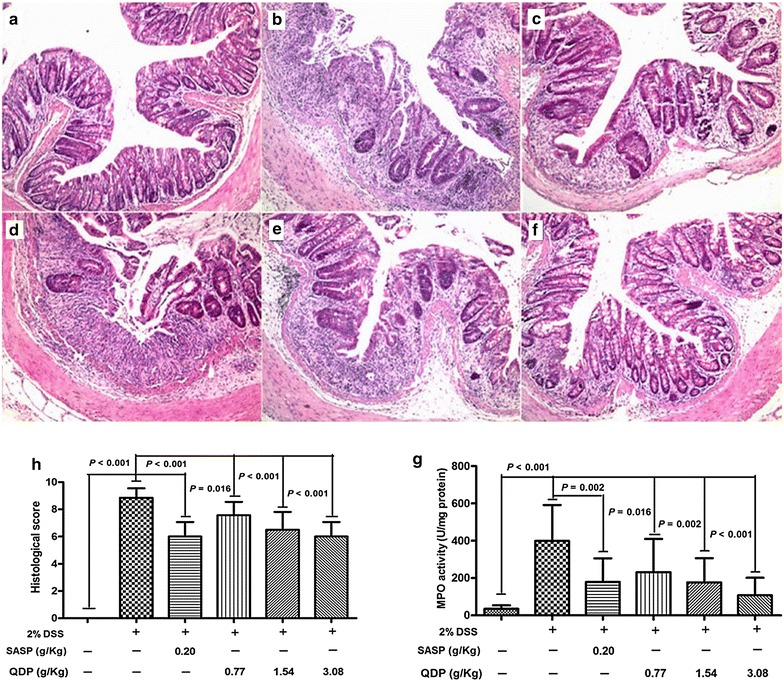
Effects of QDP on histopathological changes and MPO activity in colon of mice with DSS-induced colitis. **a** control; **b** DSS model; **c** SASP; **d** 0.77 g/kg QDP; **e** 1.54 g/kg QDP; **f** 3.08 g/kg QDP (magnification, ×100); **g** histological score; and **h** MPO activity. Colitis was induced in all groups except control group. QDP and SASP were administered to mice from day 6 to 12. On day 13, mice were sacrificed, and colonic tissue damage was evaluated by histopathological analysis (H&E staining). MPO activity was determined in colonic homogenates. Data were expressed as mean ± SD (n = 7–9)

As shown in Fig. 3, 2.0 % DSS in drinking water caused extensive colonic tissue damage, including inflammatory cell infiltration, lesion formation and crypt destruction. Mice receiving QDP treatment showed less colonic damage. Scores of the histological changes are displayed in Fig. 3g. The score was significantly higher for the DSS model group than that for the vehicle-treated control group, i.e., reducing inflammation and mucosal and crypt damage in the colon, in particular in those mice treated with medium and high doses of QDP (both *P* < 0.001). Consistent with the histological scores, colonic MPO activity was greatly increased in the DSS model group (*P* < 0.001), whereas the MPO activities in the QDP-treated groups were significantly suppressed in a dose-dependent manner (Fig. 3h**)**.

### QDP decreases infiltration of macrophages in the colon of DSS-treated mice

**Fig. 4:**
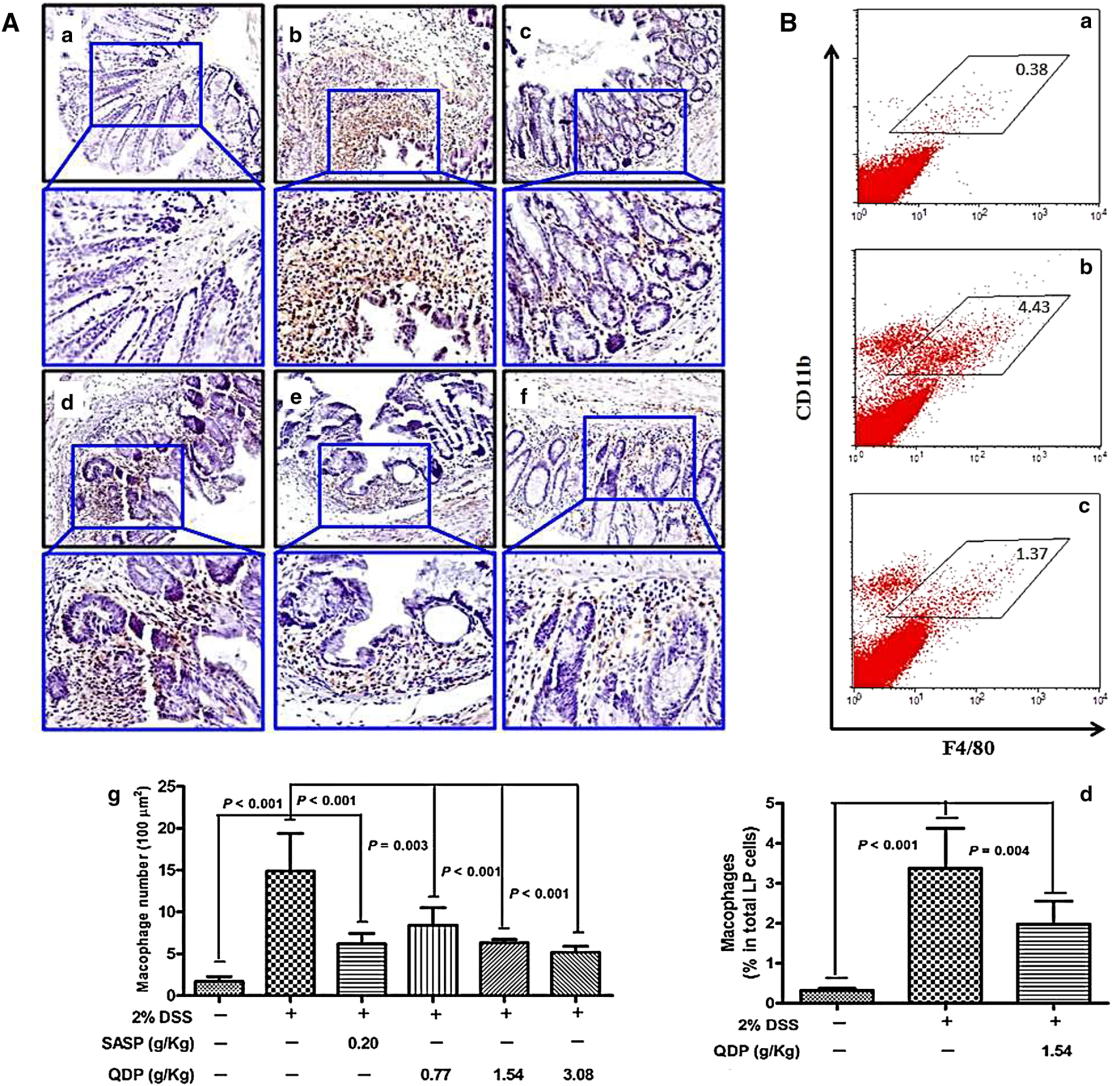
Effects of QDP on macrophage infiltration in colons of DSS-treated mice. **A** Immunohistochemical (IHC) analysis of colonic macrophages by F4/80 marker [**a**–**f** representative images (**a** control; **b** DSS model; **c** SASP; **d** 0.77 g/kg QDP; **e** 1.54 g/kg QDP; and **f** 3.08 g/kg QDP), **g** number of macrophages]; **B** flow cytometric analysis of macrophages in the colon lamina propria (LP) (**a** control; **b** DSS model; **c** 1.54 g/kg QDP; and **d** percentage of macrophages in total LP cells). Colitis was induced in all groups except control group. QDP and SASP were administered to mice from day 6 to 12. On day 13, mice were sacrificed. The colon section was evaluated by IHC analysis with F4/80 marker (n = 7–9). For flow cytometric analysis, the population of macrophage in lamina propria mononuclear cells from whole colonic tissue was determined by CD11b and F4/80 markers (n = 5 for normal control group, and n = 6 for DSS-treated groups). Data were expressed as mean ± SD

The infiltration of macrophages in the colon was analyzed with the macrophage marker F4/80. The number of macrophages in the colons of model group mice was significantly higher than in the normal group (*P* < 0.001) (Fig. 4A). Compared with the model group, there were considerably fewer macrophages in the colons of QDP-treated mice (*P* = 0.003, *P* < 0.001 and *P* < 0.001 for 0.77, 1.54 and 3.08 g/kg QDP, respectively). Similar to the results of the immunohistochemical study, the number of macrophages was increased after DSS treatment (*P* < 0.001), and QDP at 1.54 g/kg significantly decreased the recruitment of macrophages to the colon lamina propria of the DSS-treated mice (*P* = 0.004) in flow cytometry (Fig. 4B**)**.

### QDP suppresses colonic proinflammatory cytokine production and serum MCP-1 levels in DSS-treated mice

**Fig. 5:**
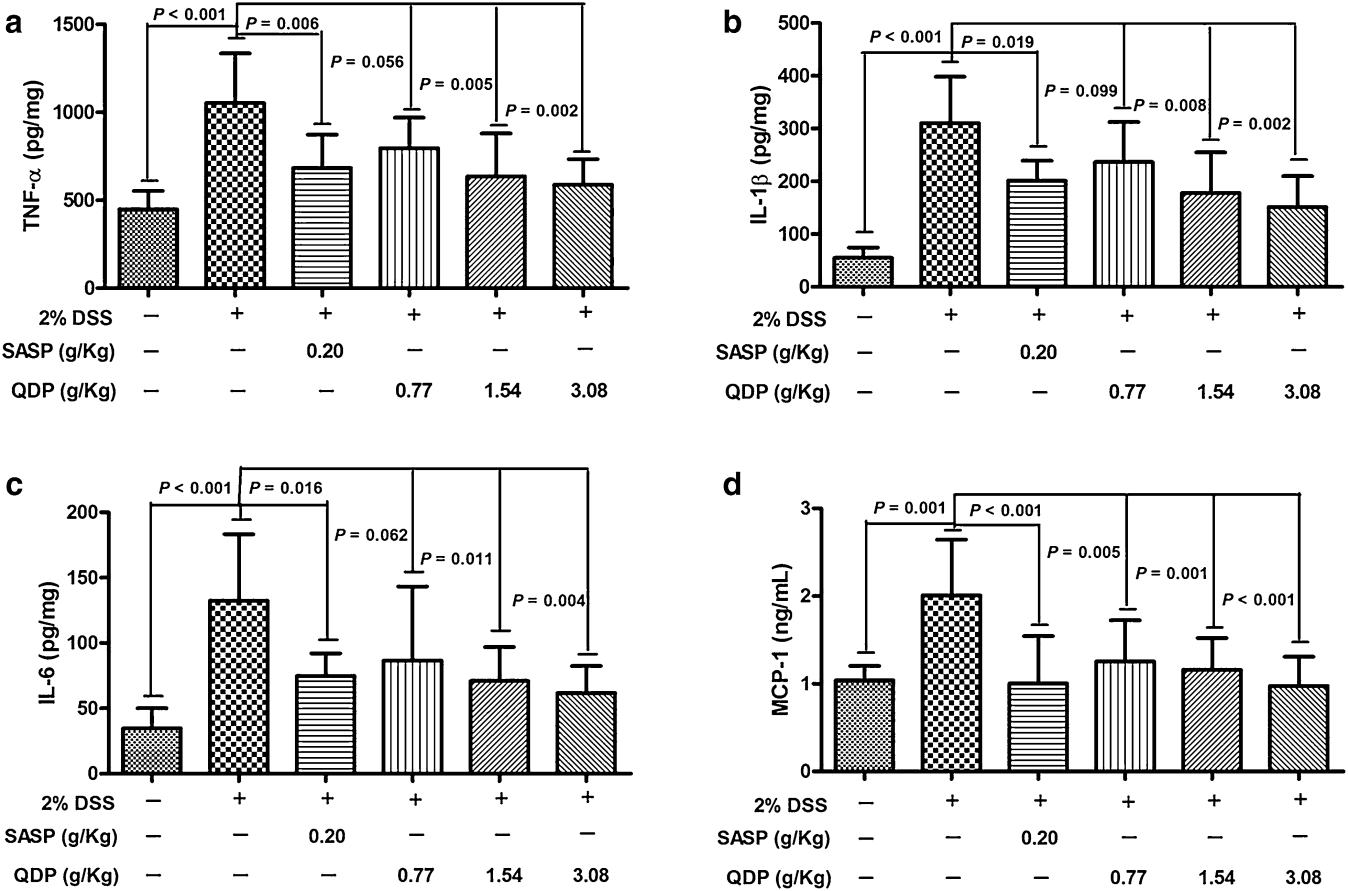
Effects of QDP on colonic production of pro-inflammatory cytokines and serum MCP-1 level in DSS-treated mice (**a** colonic TNF-α; **b** colonic IL-6; **c** colonic IL-1β; and **d** serum MCP-1). QDP and SASP were administered to mice from day 6 to 12. On day 13, mice were sacrificed, amounts of various cytokines in colonic homogenates and MCP-1 levels in serum were determined by ELISA. Data were expressed as mean ± SD (n = 7–9)

Levels of macrophage-associated proinflammatory cytokines such as TNF-α, IL-1β and IL-6 were measured in the colonic tissues to ascertain the suppressive effects of QDP on colonic macrophages. The levels of colonic TNF-α, IL-1β and IL-6 were significantly higher in the DSS model group (all *P* < 0.001), and QDP treatment significantly lowered the production of TNF-α, IL-1β and IL-6 in colonic tissue of DSS-treated mice (Fig. 5a–c). Oral administration of 2.0 % DSS in drinking water resulted in a sharp increase of MCP-1 in the serum of DSS-treated mice. Treatment with QDP greatly reduced serum MCP-1 levels in a dose-dependent manner (Fig. 5d).

### QDP inhibits LPS-induced TNF-α and IL-6 production and expression of iNOS and COX-2 in RAW264.7 cells

**Fig. 6:**
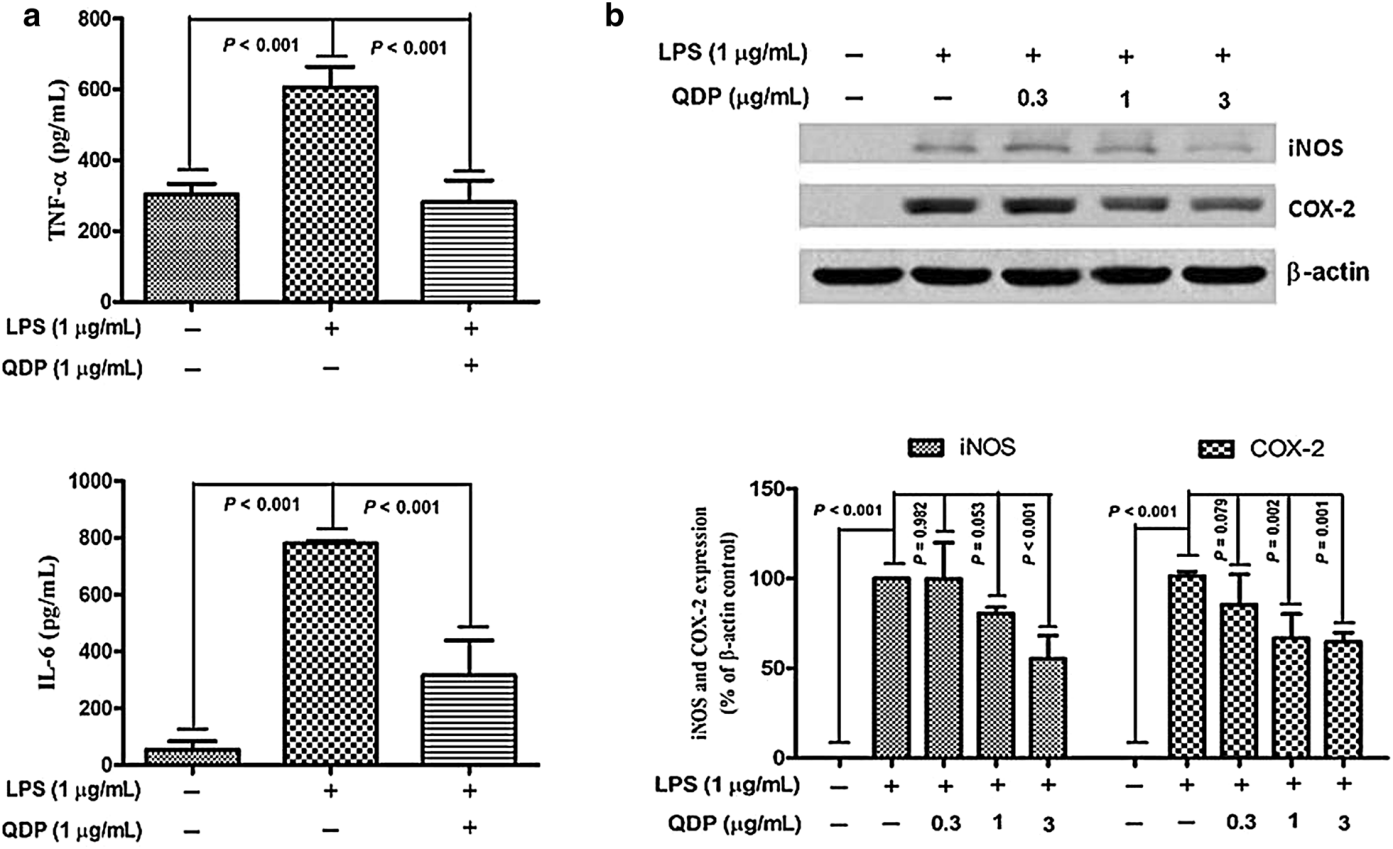
Effects of QDP on LPS-induced production of TNF-α and IL-6 and expression of iNOS and COX-2 in RAW264.7 cells. Cells were treated with QDP for 1 h, followed by continuous incubation with LPS (1 μg/mL) for 24 h. **a** Concentrations of TNF-α and IL-6 in culture medium were monitored by ELISA. **b** Cells were lysed and expression of iNOS and COX-2 were determined by Western blotting. Data represent the mean ± SD (n = 3). The images shown are representatives of three independent experiments

Macrophages are a major source of proinflammatory cytokines, including TNF-α, IL-1β and IL-6. We therefore evaluated the anti-inflammatory effect of QDP on LPS-stimulated RAW264.7 cells (murine macrophage cell line). Treatment with LPS resulted in significantly increased production of proinflammatory cytokines (by 2.0-fold for TNF-α; by 14.3-fold for IL-6) (both *P* < 0.001) in culture supernatants of RAW264.7 cells, while QDP at a concentration of 1 μg/mL significantly inhibited LPS-induced production of TNF-α and IL-6 (both *P* < 0.001) (Fig. 6a).

The activities of iNOS and COX-2 are the immediate modulators of NO and PGE_2_, respectively, produced by macrophages for the initiation and progression of IBD [33]. We investigated the effects of QDP on the expression of iNOS and COX-2 by Western blot analysis. Protein levels of iNOS and COX-2 were upregulated in response to LPS (Both *P* < 0.001), and treatment with QDP resulted in dose-dependent inhibition of iNOS and COX-2 protein expression (Fig. 6b).

### QDP blocks LPS-induced IκB-α degradation and p65 nuclear translocation

**Fig. 7:**
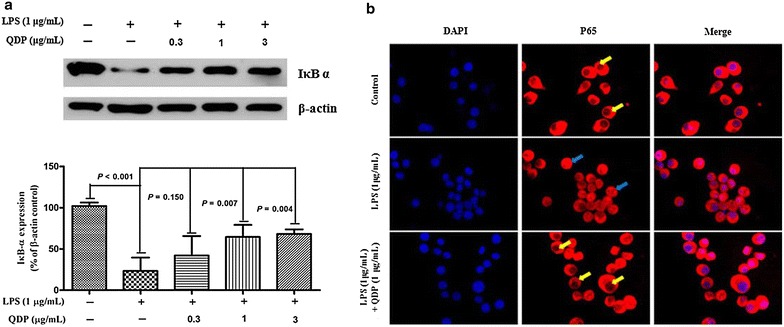
Effects of QDP on LPS-induced IκB α degradation and p65 nuclear translocation. **a** Cells were pretreated with the indicated concentrations of QDP for 1 h and incubated with LPS (1 μg/mL) for another 30 min. Cells were lysed and IκB α expression was determined by Western blot. **b** Cells were pretreated with 1 μg/mL QDP for 1 h prior to stimulation with LPS (1 μg/mL) for 1 h. The nuclear localization of p65 was determined using fluorescence microscopy after staining with DAPI, anti-p65, and FITC-labeled anti-rabbit IgG antibody (p65 sequestered in the cytoplasm were indicated by *yellow arrows* and p65 translocated into nuclear were indicated by *blue arrows)*. Data represent the mean ± SD (n = 3). The images shown are the representatives of three independent experiments

We examined the effect of QDP on LPS-induced degradation of IκB-α by Western blotting. The degradation of IκB-α after LPS treatment was inhibited by QDP in a dose-dependent manner (Fig. 7a).

Furthermore, we investigated the effect of QDP on LPS-induced p65 nuclear translocation by immunofluorescence. p65 was normally sequestered in the cytoplasm, while LPS induced p65 accumulation in the nucleus. However, pre-treatment with QDP abolished LPS-induced p65 translocation to the nucleus (Fig. 7b).

## Discussion

Oral administration of QDP suppressed colonic macrophage recruitment and activation to attenuate the severity of DSS-induced colitis in mice, as evidenced by reduced mortality, clinical manifestations, colon shortening, histological damage and colonic myeloperoxidase activity.

The DSS-induced colitis mouse model is simple and reproducible, and is commonly used to study the pathogenesis of UC, and screen potential therapeutic interventions [34]. Using this model, we found that QDP could significantly attenuate clinical symptoms of DSS-treated mice including body weight loss, stool consistency and bleeding. Furthermore, QDP significantly prevented colon shortening and colon tissue damage induced by DSS. Oral administration of QDP could attenuate the colonic MPO activity of DSS-treated mice in a dose-dependent manner.

In the inflamed gut, macrophages secrete excessive pro-inflammatory cytokines to initiate the innate immune response and the consequential adaptive immune response [14, 16]. In the present study, treatment with 2.0 % DSS resulted in an increase of macrophages in colon tissues, and oral administration of QDP could significantly suppress macrophage accumulation. The accumulated macrophages in the inflamed colon were recruited and differentiated from Ly6C^hi^ monocytes in the bloodstream, and are different from resident intestinal macrophages and represent a proinflammatory phenotype [15]. Their recruitment was mediated by MCP-1, which binds to the chemokine receptor CCR2 on circulating monocytes to attract monocytes to inflamed tissues [15, 35]. We observed that 2.0 % DSS treatment increased serum MCP-1 levels and QDP treatment could significantly reverse this increase. Accumulated colonic macrophages were activated in the context of gut inflammation, resulting in excessive secretion of proinflammatory cytokines, such as TNF-α, IL-1β and IL-6, which exacerbate IBD [35]. QDP could significantly suppress the production of TNF-α, IL-1β and IL-6 in the colons of DSS-treated mice. In RAW264.7 cells, QDP could significantly decrease LPS-induced TNF-α and IL-6 production and expression of iNOS and COX-2 by inhibiting IκB-α degradation and NF-κB p65 translocation.

SASP is used clinically for mild-to-moderate UC patients via anti-inflammatory, immunomodulatory properties or both [36]. In the present study, QDP at a dose of 1.54 g/kg exhibited an effect comparable with SASP (200 mg/kg) against DSS-induced colitis in mice. This beneficial effect was associated with its anti-inflammatory activity through suppression of colonic macrophage recruitment and activation. Therefore, QDP could be beneficial to mice, in addition to human, as a study model for developing effective pharmaceuticals to treat UC.

## Conclusion

QDP suppressed the inflammatory responses of colonic macrophages in DSS-induced UC in mice and LPS-induced RAW264.7 cells.

## Abbreviations

CMC-Na: sodium carboxymethylcellulose
COX-2: cyclooxygenase-2
DSS: dextran sulfate sodium
DAI: disease activity index
ELISA: enzyme-linked immunosorbent assay
5-ASA: 5-aminosalicylic acid
IBD: inflammatory bowel disease
IgG: immunoglobulin G
iNOS: inducible nitric oxide synthase
IκB-α: inhibitor of kappa B α
LPS: lipopolysaccharide
MPO: myeloperoxidase
NF-κB: nuclear factor kappa-light-chain-enhancer of activated B cells
PBS: phosphate buffered saline
QDP: *Qing-dai* powder
UC: ulcerative colitis
UPLC-QTOF-MS: ultra-performance liquid chromatography quadrupole time of flight mass spectrometry
SASP: sulfasalazine
BPI: bits per inch
IHC: immunohistochemistry
